# Favorable Evaluations of Black and White Women’s Workplace Anger During the Era of #MeToo

**DOI:** 10.3389/fpsyg.2021.594260

**Published:** 2021-02-25

**Authors:** Kaitlin McCormick-Huhn, Stephanie A. Shields

**Affiliations:** ^1^William S. Boyd School of Law, University of Nevada, Las Vegas, Las Vegas, NV, United States; ^2^Department of Psychology, The Pennsylvania State University, University Park, PA, United States

**Keywords:** gender stereotyping, stereotypes, emotion, historical context, workplace, anger, cultural events, #MeToo

## Abstract

Researchers investigating gender and anger have consistently found that White women, but not White men, are evaluated unfavorably when experiencing anger in the workplace. Our project originally aimed to extend findings on White women’s, Black women’s, and White men’s workplace anger by examining whether evaluations are exacerbated or buffered by invalidating or affirming comments from others. In stark contrast to previous research on gender stereotyping and anger evaluations, however, results across four studies (*N* = 1,095) showed that both Black and White women portrayed as experiencing anger in the workplace were evaluated *more favorably* than White men doing so. After Study 1’s initial failure to conceptually replicate, we investigated whether perceivers’ evaluations of women’s workplace anger could have been affected by the contemporaneous cultural event of #MeToo. Supporting this possibility, we found evaluations were moderated by news engagement and beliefs that workplace opportunities are gendered. Additionally, we found invalidating comments rarely affected evaluations of a protagonist yet affirming comments tended to favorably affect evaluations. Overall, findings suggest the need for psychologists to consider the temporary, or perhaps lasting, effects of cultural events on research outcomes.

## Introduction

Research on gender and anger typically has shown that White women, but not White men, are perceived negatively when described as experiencing anger in the workplace (Lewis, 2000; Brescoll and Uhlmann, 2008; Gibson et al., 2009). In their 2008 study, Brescoll and Uhlmann found that White women are perceived as less competent, are conferred lower status and salary, and are perceived as higher in dispositional emotionality than White men when angry at work. In the studies that follow, we sought to conceptually replicate the findings of Brescoll and Uhlmann (2008) and to extend those findings by examining (a) how Black women’s anger is perceived relative to White men’s and (b) whether evaluations of anger are exacerbated or buffered by social information from others. Below, we review literature on the perceptions of anger and its appropriateness, perceptions of White women’s anger in the workplace, perceptions of Black women’s anger and work behaviors, influences of others on appropriateness judgments, influences of sociohistorical context, and the present studies’ sociohistorical context of #MeToo and gender equity in the workplace.

### Perceptions of Anger and Its Appropriateness

Anger is an emotion that is evoked when one feels they have been unfairly wronged (e.g., Lerner, 1985; Rozin et al., 1999; Shields, 2002) and is accompanied by an agentic action tendency to do something to change the situation (e.g., Frijda et al., 1989). Although anger is often thought of as a “negative” emotion, in the workplace context specifically, anger is not inherently negative (Geddes and Callister, 2007; see also Averill, 2011). Expressing anger can be thought of as claiming respect (Frye, 1983), and expressing the objective of change (Lorde, 1984).

Judgments about emotion appropriateness are open to perceivers’ interpretation (Shields, 2005) and are affected by social group stereotypes (e.g., gender and race; e.g., Hall and Livingston, 2012). Judging someone’s anger as inappropriate has the further damaging consequence of questioning a person’s legitimacy (Lorde, 1984; Campbell, 1994; Warner and Shields, 2009a). Thus, for whom anger is deemed appropriate may tell us something about societal power structures: people’s evaluations of certain social group members’ anger as inappropriate may reveal people’s beliefs about who is entitled to feel their individual rights have been violated and who is entitled to seek justice.

### Perceptions of White Women’s Anger in the Workplace

In general, people believe women’s anger is less common and less appropriate than men’s anger (e.g., Fabes and Martin, 1991; Sharkin, 1993; Plant et al., 2000); although few differences in women and men’s actual experiences and expressions of anger are found (e.g., Averill, 1983). Unlike White men’s anger, White women’s anger is often believed by others to be an emotional response caused by women’s stereotypically emotional dispositions, rather than believed to be an expression of anger caused by the situation (e.g., Brescoll and Uhlmann, 2008; Barrett and Bliss-Moreau, 2009). In the workplace, in particular, White women, but not White men, have been evaluated unfavorably for experiencing anger (Lewis, 2000; Brescoll and Uhlmann, 2008; Gibson et al., 2009). For instance, although expressing anger in the workplace can result in gaining status for White men, anger expression for White women can lead to decreased status (e.g., Ragins and Winkel, 2011). Overall, research on anger in the workplace supports the prediction that White women’s anger will be evaluated as less appropriate than White men’s anger.

### Perceptions of Black Women’s Anger and Work Behaviors

In contrast, the relation between Black women’s anger and status in the workplace has so far yielded mixed results. For example, Black women’s workplace anger relative to White men’s reveals stereotypes of Black women as overly angry (e.g., Harris-Perry, 2011). Yet agentic behaviors that share action tendencies with anger displays (e.g., Frijda et al., 1989) do not appear to disadvantage Black women. For example, Black women leaders, like White men leaders, did not face backlash for agentic behaviors (Livingston et al., 2012) and were favorably evaluated when enacting agentic behaviors in leadership roles (Livingston et al., 2012). Further, Black women faced especially harsh penalties for failure, the antithesis of agenticism (Rosette and Livingston, 2012). For these reasons, to conceptually replicate and extend findings of Brescoll and Uhlmann (2008), we focused on perceptions of Black and White women’s anger, relative to perceptions of White men’s anger.

### Influences of Others on Emotion Appropriateness Judgments

Research on whose emotion is perceived as appropriate reveals a pattern of inequity for people experiencing emotion of particular social group memberships (e.g., Power et al., 2010; Hall and Livingston, 2012). As the research reviewed above suggests, perceivers (i.e., research participants) often determine appropriateness and do so in a way that maintains social power relationships by relying on social group stereotypes to discredit or bolster a protagonist (i.e., a person experiencing an emotion). Although perceivers ultimately evaluate a protagonist’s emotion appropriateness, other people in the social environment may play a role in influencing perceivers’ judgments of a protagonist’s appropriateness as well.

To extend our conceptual replication of Brescoll and Uhlmann (2008), we examined whether an invalidator or an affirmer (i.e., a person who comments on a protagonist’s emotion) could exacerbate or buffer perceivers’ judgments of a protagonist. If so, drawing on expectation states theory, the social status of an invalidator or affirmer may also influence perceptions of a protagonist’s emotional appropriateness, such that comments on another’s emotion by an invalidator or affirmer with relative structural power (e.g., White man relative to White woman) could have ripple effects on perceivers’ judgments (Ridgeway, 2006). For example, high status people are accorded what Correll et al. (2017) term a “status advantage.” That is, when quality of a person’s contribution was uncertain, research participants ascribed higher quality of the contribution to higher status individuals (Correll et al., 2017). Thus, we tested whether an invalidator or affirmer, especially someone associated with higher status through their gender and race social group memberships, might exacerbate or buffer perceptions of a protagonist’s anger by providing an invalidating or affirming comment. In summary, we evaluated whether invalidators or affirmers who made invalidating or affirming comments about someone’s anger affected perceivers’ evaluations of a protagonist, and if the social group memberships of the angry party or of the invalidators affected perceivers’ judgments differently.

### Influences of Sociohistorical Context

Although research on stereotypes about gender and emotion suggests these stereotypes tend to remain stable over time (e.g., Shields et al., 2018), the sociohistorical context could also affect perceivers’ judgments of anger appropriateness. Indeed, the COVID-19 pandemic that currently is sweeping the world is a reminder that historic and cultural events can have a broad effect on psychological phenomena (e.g., Plant et al., 2009; Sawyer and Gampa, 2018; Yates and Okimoto, 2019). Psychologists have been most engaged with investigating the direct effects of these events (e.g., Rudman et al., 2013). For example, Rudman et al. (2013) found participants showed negative implicit attitudes toward a green politician before experiencing Hurricanes Irene and Sandy yet showed positive implicit attitudes toward such a politician when attitudes were tested after the hurricanes.

One theory of attitude change, “the normative window,” suggests prejudicial attitudes toward most social groups are not stable (Crandall et al., 2018). Rather, the normative window theory suggests prejudice toward a social group reflects prejudicial attitudes during a “window of time in which social norms are shifting toward equal treatment…but for which the entire process has not yet been completed, and for which complete social agreement about the status of the group has not yet been achieved” (Crandall et al., 2013, p. 56). For instance, a shift in social norms pertaining to the acceptability of prejudice was found after the election of Donald Trump, with prejudice toward social groups that were targeted by the Trump campaign in 2016 (e.g., immigrants, disabled people, and Muslims) rated as more acceptable than it was pre-election (Crandall et al., 2018). Thus, the normative window theory supports the idea that as social norms about social groups shift, even temporarily, attitudes can shift in line with changing norms. Specific to gender, attitudes about gender in the United States can shift with women’s movement activity (Banaszak and Ondercin, 2016), with national partisan policy change (Kellstedt et al., 2010), and during particular decades in history (e.g., Donnelly et al., 2016; Shu and Meagher, 2017; Lee et al., 2018).

Attitude change can also signal a relatively stable change, such as when something that a society previously considered as a preference acquires a moral dimension (Rozin, 1999). For instance, the process of moralization has occurred in the United States for attitudes toward cigarette smoking. In particular, moralization appears to occur for behaviors that are health relevant and to occur in Protestant cultures that emphasize self-control (Rozin, 1999). Although it is most common for behaviors to become negatively moralized, the moral dimensions of behavior can also shift toward neutral, for instance, in the case of alcohol in the United States shifting from Prohibition-era attitudes to today’s attitudes (Rozin, 1999). Therefore, a change in social norms or attitudes may reflect a temporary change, or, as in the case of moralization, may suggest a change that will last for decades.

Events that are occurring only in the backdrop of our research may also color the research landscape, having temporary, or perhaps even lasting, influence on what we believe to be established patterns of results. For example, during Barack Obama’s presidential candidacy, Plant et al. (2009) did not replicate expected patterns of implicit anti-Black bias. Their unexpected findings served as a springboard for “the Obama Effect,” the finding that participants’ accessibility of Obama as a counter-stereotypic Black exemplar was associated with lower than typical rates of anti-Black implicit bias (Plant et al., 2009), with individual difference factors (e.g., anti-prejudice motivations) and contextual factors (e.g., media portrayals) affecting the strength of the effect (Rivera and Plant, 2016).

We conducted the present research during winter and spring of 2018, a time period in which the #MeToo movement catalyzed widespread media focus on issues of gender discrimination, sexual assault, and sexual harassment in the workplace (e.g., Johnson and Hawbaker, 2021). The broad social movement occurring in the backdrop of our research, specifically #MeToo and related concerns, may have affected perceptions of women’s anger in the workplace. Therefore, the goals of our conceptual replication expanded during our research process to include the measurement of possible effects of this sociohistorical context on our findings.

### The Sociohistorical Context of the Present Studies: #MeToo and Gender Equity in the Workplace

Much of the #MeToo news coverage involved specific mention of women’s anger and the justified nature of such anger (e.g., Garber, 2017). Indeed, this shift in perception of women’s anger was emphasized in news coverage of the #MeToo movement as well. Garber (2017), for instance, who described actor Uma Thurman’s labeling of herself as angry and as waiting to be less angry to speak about Harvey Weinstein, wrote, “A celebrity, expressing anger that did not bother to hide itself beneath a gauze of easy pleasantry. That anger, going viral. It was a weekend that witnessed that rarest of events: the American public, applauding a furious woman.” Common themes in media pieces such as this one, led us to consider news engagement and later, beliefs in workplace opportunities as gendered, as moderators that might have affected findings that emerged in our first study.

#MeToo was perhaps the most visible, but not the only discussion of women’s experiences in the workplace occurring at the time. For example, pay inequity was also receiving much media attention (e.g., Calfas, 2018), as well as discussions of resistance reported by men to mentor women in the workplace in the aftermath of #MeToo (SurveyMonkey, 2018). We reasoned that, beyond engaging with news about gender discrimination and harassment in the workplace, we should measure the degree to which people also endorsed ideas that women experience bias and limited opportunity relative to men in the workplace. The specific construct of belief in workplace opportunities as gendered (BWOG) was created to capture beliefs about gender dynamics at work during #MeToo as a moderator as well.

## Research Overview

In the present studies, we investigated evaluations of White women’s (Studies 1a and 1b) and Black women’s (Studies 2 and 3) anger in the workplace, each relative to White men’s. The intersectional positions (i.e., gender and race) we selected for comparison were derived from our research questions (Warner, 2008). In Studies 1a and 1b, we chose to compare evaluations of White women and White men to conceptually replicate the design in Brescoll and Uhlmann (2008). For Studies 2 and 3, we chose to compare evaluations of Black women and White men as an extension of the design in Brescoll and Uhlmann (2008). We chose this comparison because of mixed support in the literature for predictions of the comparison between Black women’s anger and White men’s anger. In particular, Black women leaders, like White men leaders, are favorably evaluated when enacting behaviors similar to anger displays (Livingston et al., 2012), yet Black women are also stereotyped as overly angry (e.g., Harris-Perry, 2011). We chose not to compare Black women and White women because we were most interested in evaluations of anger relative to White men, a social group for whom experiencing anger in the workplace can lead to status (e.g., Ragins and Winkel, 2011). And we chose not to compare Black women to Black men because of unique stereotypes about Black men’s anger (e.g., Jackson and Wingfield, 2013) and a lack of demonstrated connection between Black men’s anger at work and status gains.

We also examined the effects of invalidation (Studies 1a, 1b, and 2) and affirmation (Study 3). Additionally, when we did not conceptually replicate findings of White men’s anger being evaluated more favorably than White women’s in Study 1a, we identified a potential explanation for the failure to replicate and tested that possibility while continuing with the original investigation of perceptions of women’s anger in the workplace. Therefore, we examined effects of cultural events through moderators of news engagement (Study 1b) and BWOG (Studies 2 and 3).

Across studies, we measured emotion appropriateness including both appropriateness of emotion type and appropriateness of emotion intensity. We were especially interested in emotion appropriateness because appropriateness judgments are often contested and affected by social group stereotypes (Shields, 2005). We reasoned that emotion appropriateness judgments might also be affected by social information in the form of invalidating and affirming comments from others. We also measured variables from Brescoll and Uhlmann (2008) to test for conceptual replication. These variables included dispositional emotionality, a typically gendered judgment that anger or other emotion is personality-based rather than due to the situation (e.g., Barrett and Bliss-Moreau, 2009), and consequential workplace outcomes of competence, conferred status, and conferred salary. In addition, in Study 1a, we measured authenticity as a control variable to ensure protagonists’ anger did not appear dishonest or unfelt.

## Study 1a: Anger Invalidation

Study 1a aimed to conceptually replicate findings that suggest White women’s anger is evaluated less favorably than White men’s (conditions with no invalidators). To extend findings, we examined the possibly exacerbating role of an invalidator’s verbal invalidation on appropriateness evaluations (Shields, 2005). We also examined possible interacting effects of protagonist and invalidator intersectional positions (varied by gender and race). In line with research on expectation states theory and status advantage (e.g., Correll and Ridgeway, 2006), we predicted that invalidating comments from White men would have an especially damaging effect on perceivers’ evaluations of angry White women.

### Method

#### Participants

Undergraduate psychology students in the United States participated online, remotely through a university-hosted site for course credit. The final sample had 234 people after exclusions (see Table 1 for demographics and Table 2 for exclusions for this and subsequent studies; see Supplementary Material for sample size determination for this and subsequent studies).

**Table 1:** Participant demographics across studies.

	Study 1a (*N* = 234)	Study 1b (*N* = 268)	Study 2 (*N* = 297)	Study 3 (*N* = 296)
**Gender**				
Women	125	135	160	168
Men	108	130	133	125
Transgender	0	0	0	2
Prefer not to say	1	3	4	1
Age *M* (*SD*)	19.14 (1.38)	35.97 (10.85)	37.92 (12.61)	35.83 (11.37)
Range	18–27	18–76	19–80	19–77
**Race/ethnicity**				
% Asian/Asian-American	10.3	7.5	9.1	4.7
% Black	3.4	6.7	9.8	6.1
% Latina/o/x	4.7	7.1	4.4	7.8
% Middle Eastern	0.9	0	0	1
% multiracial	3.8	3	2.4	1.7
% Native American or Alaska Native	0	0	0.3	0.7
% other	1.3	0	1.6	0.3
% White	75.6	75.7	72.4	77.7
Years of work experience *M* (*SD*)	1.31 (1.74)	16.00 (1.63)	17.57 (12.18)	16.77 (10.90)
Range	0–12	0–50	1–55	0–50
**Experience working in an office**				
% yes	22.6	83.2	87.2	86.5
% no	76.1	16	12.1	13.2
% unsure	1.3	0.7	0.7	0.3
Political ideology *M* (*SD*)	--	3.46 (1.61)	3.62 (1.63)	3.43 (1.73)

Political ideology was measured with a single item on a scale of (1) Very Liberal to (7) Very Conservative.

**Table 2:** Participant exclusions across studies.

	Study 1a (*n* = 35)	Study 1b (*n* = 83)	Study 2 (*n* = 126)	Study 3 (*n* = 85)
**Reason for exclusion**				
Failed comprehension check	10	15	18	15
Failed attention check	6	12	4	10
Completed study on phone	2	13	13	21
Failed characters’ gender or race manipulation checks	17	43	91	39

Manipulation checks were open-ended prompts to identify the race and gender of the main character and/or invalidator/affirmer at the end of the study. Normative exclusion rate due to failed manipulation checks for Amazon Mechanical Turk samples, 19% (e.g., Goodman et al., 2013; Salerno et al., 2019).

#### Materials

**Figure 1:**
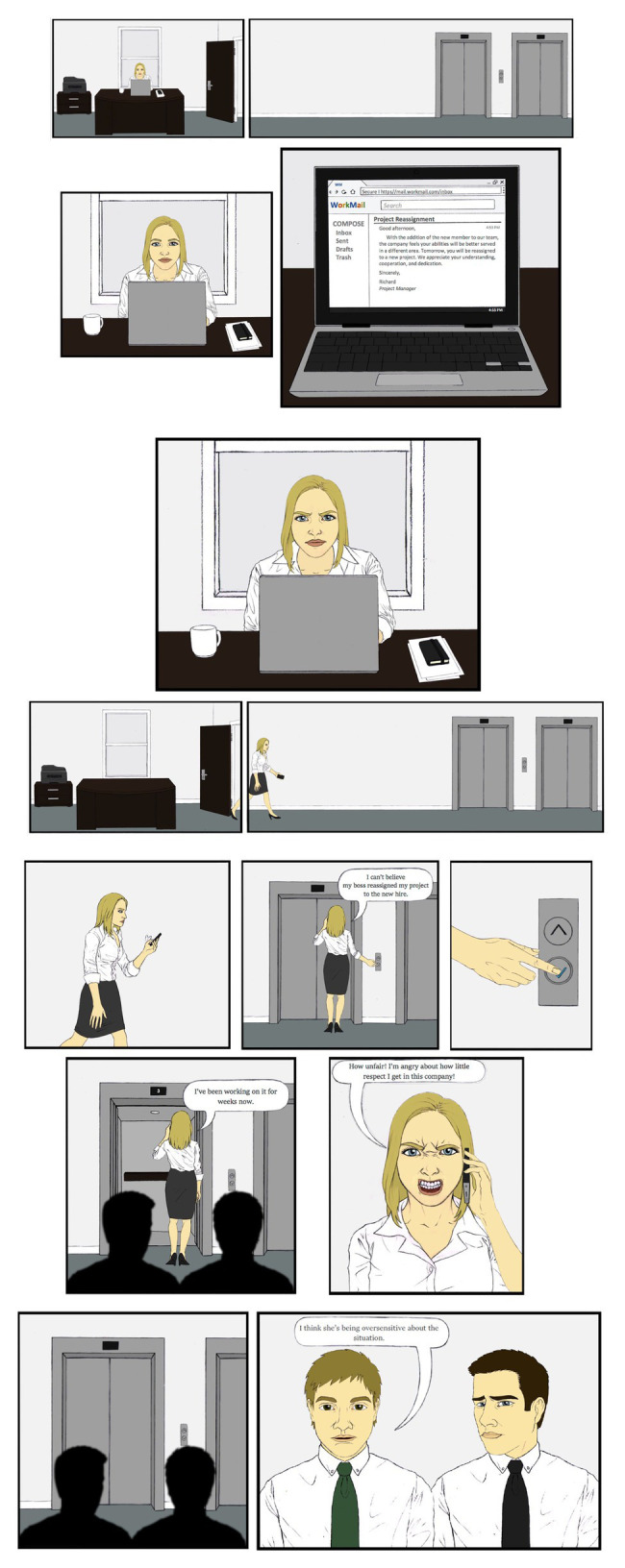
An emotion storyboard used in Study 1a. To create a situation to examine stereotyping and invalidation, the protagonist’s dialogue, expressions, and self-labeling made the fact that they were angry unambiguous, yet the reason for the protagonist’s reassignment was intentionally ambiguous.

Professionally drawn illustrated stories modeled in a graphic novel-like format were used to depict characters and their emotional responses in a workplace encounter (Figure 1). This method, “the emotion storyboard method” (McCormick-Huhn and Shields, under review), was used to portray protagonists and invalidators. Character race and gender (i.e., intersectional position; see Supplementary Material) was manipulated through identifiable characteristics (e.g., hair length, clothing type, and skin tone). Characters were piloted beforehand to ensure participants recognized the race and gender of the characters with at least 80% consensus. Character anger expressions were also piloted (see Supplementary Material). Sample materials are provided within the manuscript, and all measures are provided in Supplementary Material. The complete set of experimental materials is available from the corresponding author upon request.

#### Measures

Participants responded to the following measures on 7-point Likert scales ranging from 1 (Strongly disagree) to 7 (Strongly agree). Scale reliabilities, means, and standard deviations across studies are detailed in Table 3 (all items provided in Supplementary Material).

*Appropriateness of emotion type* (adapted from Warner and Shields, 2009b).

**Table 3:** Reliabilities, means, and standard deviations of measures across studies.

Scale	Study 1a	Study 1b	Study 2	Study 3
**Appropriateness of emotion type**				
*M* (SD)	4.97 (0.96)	4.85 (1.33)	4.94 (1.25)	5.27 (1.16)
Cronbach’s *α*	0.72	0.84	0.83	0.80
**Appropriateness of emotion intensity**				
*M* (SD)	4.71 (1.15)	4.19 (1.65)	4.49 (1.48)	4.80 (1.49)
Cronbach’s *α*	0.83	0.93	0.91	0.91
**Dispositional emotionality**				
*M* (SD)	3.98 (0.76)	4.14 (0.90)	4.01 (0.97)	3.79 (1.01)
Cronbach’s *α*	0.65	0.71	0.77	0.79
**Authenticity**				
*M* (SD)	5.43 (0.94)	--	--	--
Cronbach’s *α*	0.74	--	--	--
**Competence**				
*M* (SD)	4.52 (1.15)	4.60 (1.09)	--	--
Spearman’s rho, *p*	0.73, <0.001	0.84, <0.001	--	--
**Conferred status**				
*M* (SD)	3.95 (1.07)	4.03 (1.18)	4.22 (1.10)	4.33 (1.16)
Cronbach’s *α*	0.88	0.92	0.89	0.90
**Conferred salary (*$*)**				
*M* (SD)	57,484.99 (18,438.23)	44,893.18 (14,023.43)	46,291.02 (15,247.25)	47,237.72 (14,980.10)
Cronbach’*s* α	--	--	--	--
**News engagement**				
*M* (SD)	--	4.84 (1.38)	--	--
Cronbach’s *α*	--	0.72	--	--
**Social desirability**				
*M* (SD)	--	0.47 (0.25)	--	--
Cronbach’s *α*	--	0.78	--	--
**BWOG**				
*M* (SD)	--	--	4.90 (1.53)	5.20 (1.45)
Cronbach’s *α*	--	--	0.94	0.92

BWOG indicates beliefs in workplace opportunities as gendered.

Appropriateness of emotion type was measured with four items, e.g., “The main character’s emotions were exactly the kinds that were called for.”

*Appropriateness of emotion intensity* (adapted from Warner and Shields, 2009b).

Appropriateness of emotion intensity was measured with five items, e.g., “The main character was too emotional” (reverse-coded).

*Dispositional emotionality* (adapted from McCormick-Huhn et al., in preparation and Brescoll and Uhlmann, 2008).

Dispositional emotionality was measured with six items adapted from two scales, e.g., “In general, how likely is the main character to overreact?; The main character became angry because of his or her personality.”

*Authenticity* (adapted from Zawadzki et al., 2013).

Authenticity was measured with four items, e.g., “How genuine was the main character’s emotion?” Authenticity was included as a control variable and was unaffected by experimental variations. Results for authenticity are reported in Supplementary Material.

*Competence* (adapted from Brescoll and Uhlmann, 2008).

Competence was measured with two items, e.g., “How skilled is the main character?”

*Conferred status* (adapted from Tiedens, 2001).

Conferred status was measured with four items assessing how much status the protagonist deserved, e.g., “How much power does the main character deserve?”

*Conferred salary* (adapted from Brescoll and Uhlmann, 2008).

Salary conferral was measured with one, open-ended item, which asked participants for the yearly salary amount they would pay the main character.

#### Procedure

Undergraduate students were randomly assigned to one of six conditions in a 2 (protagonist: White woman vs. White man) × 3 (invalidator: White woman vs. White man vs. none/control) between-subjects design. The study was conducted online. Participants viewed a picture of the protagonist and then read an emotion storyboard. The storyboard depicted the protagonist learning their project had been reassigned. Two coworkers overhear the protagonist angrily recounting the situation to someone on the phone. In invalidator conditions, once the protagonist leaves, one coworker, the invalidator, tells the other they think the protagonist is being oversensitive. Participants answered questions about the protagonist’s emotion, imagined themselves as the protagonist’s supervisor to answer questions about competence, status, and salary, and completed a comprehension check, an attention check, manipulation checks, and demographics.

### Results

Between-subjects ANOVAs were conducted, using SPSS Statistics (IBM Corp, 2017), for each of the measures. See Table 4 for means and standard deviations of protagonist effects.

**Table 4:** Study 1a means (standard deviations).

	Main character intersectional position	
	White woman	White man
Appropriateness of emotion type	5.15 (0.95)^a^	4.81 (0.94)^b^
Appropriateness of emotion intensity	4.89 (1.12)^a^	4.53 (1.16)^b^
Dispositional emotionality	3.83 (0.84)^a^	4.12 (0.65)^b^
Competence	4.54 (1.25)^a^	4.49 (1.05)^a^
Status	4.14 (1.03)^a^	3.78 (1.09)^b^
Salary	$58,317.61 ($18,895.50)^a^	$56,724.14 ($18,058.64)^a^

Different superscripts (i.e., a and b) indicate the two main character conditions significantly differed from one another on the variables of interest (by at least *p* < 0.05).

Appropriateness of emotion type differed by protagonist, *F*(1, 228) = 7.77, *p* = 0.006, *d* = 0.36, 95% CI [0.11, 0.63], such that the White woman was rated more appropriate in emotion type than the White man. Appropriateness of emotion type did not differ based on invalidator (*p* = 0.542) or interaction of protagonist and invalidator (*p* = 0.819).

Appropriateness of emotion intensity differed by protagonist, *F*(1, 228) = 5.52, *p* = 0.020, *d* = 0.32, 95% CI [0.05, 0.57], such that the White woman was rated more appropriate in emotion intensity than the White man. Appropriateness of emotion intensity did not differ based on invalidator (*p* = 0.051) or interaction of protagonist and invalidator (*p* = 0.987).

Dispositional emotionality differed by protagonist, *F*(1, 228) = 7.90, *p* = 0.005, *d* = 0.39, 95% CI [0.11, 0.63], such that the White man was rated as higher in dispositional emotionality than the White woman. Dispositional emotionality did not differ based on invalidator (*p* = 0.239) or the interaction of protagonist and invalidator (*p* = 0.426).

Competence did not differ by protagonist (*p* = 0.751), invalidator (*p* = 0.848), or the interaction of protagonist and invalidator (*p* = 0.239).

Conferred status differed by protagonist, *F*(1, 228) = 6.72, *p* = 0.010, *d* = 0.34, 95% CI [0.08, 0.60], such that the White woman was conferred more status than the White man. Conferred status did not differ based on invalidator (*p* = 0.501) or the interaction of protagonist and invalidator (*p* = 0.889).

Conferred salary did not differ by protagonist (*p* = 0.722), invalidator (*p* = 0.319), or the interaction of protagonist and invalidator (*p* = 0.072; see Supplementary Material for salary exclusions).

### Discussion

Surprisingly, protagonist effects directly contrasted with predictions and prevailing patterns in previous research: White women were rated more appropriate in emotion type and emotion intensity, lower in dispositional emotionality, and more deserving of status than White men. Competence and conferred salary did not differ based on protagonist. Predictions that a comment from an invalidator would affect judgments, and that intersectional positions of invalidators and protagonists would interact to affect judgments, were unsupported across all measures.

## Study 1b: the Role of News Engagement

Study 1b attempted to rule out that findings were an artifact of the sample. Study 1b was identical to Study 1a, but conducted with Amazon Mechanical Turk workers, a population with more work experience than undergraduates (e.g., Levay et al., 2016). Alternatively, findings may have been affected by the study’s broader context, specifically by the #MeToo movement and the cultural conversation about gender discrimination, sexual assault, and sexual harassment in the workplace that it inspired. Given the salience of contemporaneous cultural events to the topic under investigation, we predicted Study 1b would replicate Study 1a results of White women being evaluated more favorably than White men, and that participants high in news engagement would drive this pattern. We also controlled for effects of participant political ideology and tendency toward socially desirable responding. Participant political ideology was one factor that might have affected findings during this cultural moment because explicit reports of perceiving the #MeToo movement favorably varied by political orientation (Bucknell’s Public Policy Institute, 2018). Additionally, it was possible that participants exposed to a large amount of gender-relevant news responded to measures about White women more positively than in the past due to a tendency to respond in a socially desirable manner when asked to evaluate a woman in the workplace.

### Method

#### Participants

Workers from Amazon Mechanical Turk (MTurk) in the United States participated in the study online. The final sample had 268 people.

#### Materials

Materials were the same as Study 1a.

#### Measures

Measures from Study 1a were used and the following were added (see Table 3 for scale reliabilities, means, and standard deviations):

*News engagement* (created for this study).

News engagement was measured with a three-item scale (“How frequently do you read news articles?”; “To what extent are you familiar with the #MeToo movement?”; and “How often have you come across news articles about gender discrimination in the workplace?”). Participants responded on 7-point Likert scales ranging from 1 (Strongly disagree) to 7 (Strongly agree).

Political ideology.

Participants responded to a single item ranging from (1) Very Liberal to (7) Very Conservative (see Table 1).

*Social desirability* (Reynolds, 1982).

Social desirability was measured with a 13-item scale, e.g., “I am always courteous, even to people who are disagreeable.” Participants responded by selecting if the described behavior was “true” or “false” of themselves.

#### Procedure

MTurk workers participated online. The procedure and conditions were the same as Study 1a, except, participants were also told that there were questions about their own behaviors and personality traits.

### Results

**Figure 2:**
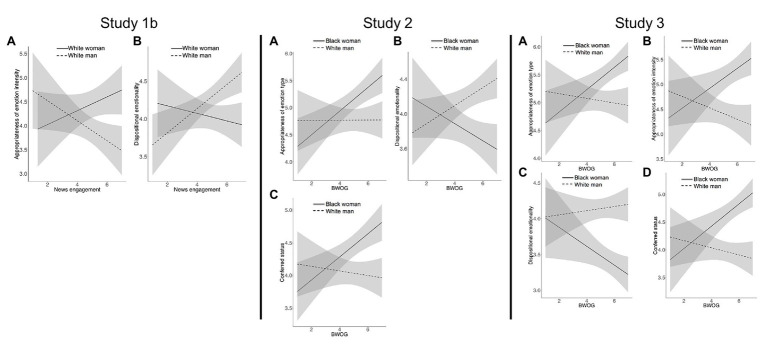
Moderated effects across Studies 1b, 2, and 3. Left panel depicts Study 1b simple slopes for appropriateness of emotion intensity **(A)** and dispositional emotionality **(B)** at levels of news engagement. Values on the y-axes and x-axes were on a scale of 1 (Strongly disagree) to 7 (Strongly agree). Value for news engagement one standard deviation below the mean: 3.46, value for news engagement one standard deviation above the mean: 6.22. Error bands indicate 95% confidence intervals. Results did not differ if covariates were included in analyses so simple slopes reported are from analyses without covariates. Middle panel depicts Study 2 simple slopes for appropriateness of emotion type **(A)**, dispositional emotionality **(B)**, and conferred status **(C)** at levels of beliefs in work opportunities as gendered (BWOG). Values on the y-axes and x-axes were on a scale of 1 (Strongly disagree) to 7 (Strongly agree). Value for BWOG one standard deviation below the mean: 3.37, value for BWOG one standard deviation above the mean: 6.43. Error bands indicate 95% confidence intervals. Right panel depicts Study 3 simple slopes for appropriateness of emotion type **(A)**, appropriateness of emotion intensity **(B)**, dispositional emotionality **(C)**, and conferred status **(D)** at levels of beliefs in work opportunities as gendered (BWOG). Values on the y-axes and x-axes were on a scale of 1 (Strongly disagree) to 7 (Strongly agree). Value for BWOG one standard deviation below the mean: 3.75, value for BWOG one standard deviation above the mean: 6.65. Error bands indicate 95% confidence intervals.

For Study 1b and subsequent studies, we conducted regression analyses using SPSS Statistics (IBM Corp, 2017). For each measure, we examined whether the effect of White woman protagonist would be moderated by news engagement, using two regression models. Model 1 regressed measures on White woman protagonist (dummy coded), the contrast of being invalidated to not being invalidated (coded: no invalidation = −2, invalidated by White woman = 1, and invalidated by White man = 1), the contrast of being invalidated by a White man to being invalidated by a White woman, the interaction of invalidator and protagonist, political ideology, and tendency toward socially desirable responding. In Model 2, we added the White woman protagonist x news engagement interaction, as well as news engagement on its own to distinguish independent effects of this variable on measures (see Supplementary Material for full regression table and complete variable coding information). For significant White woman protagonist x news engagement interactions, we estimated simple slopes for news engagement one standard deviation above and below the mean. Simple slopes are depicted in Figure 2.

In Model 1, White woman protagonist predicted appropriateness of emotion type (*b* = 0.46, 95% CI [0.14, 0.77], *p* = 0.004). The contrast of being invalidated to not being invalidated also predicted appropriateness of emotion type (*b* = −0.12, 95% CI [−0.23, −0.01], *p* = 0.026). In Model 2, the White woman protagonist x news engagement interaction did not predict appropriateness of emotion type (*b* = 0.17, 95% CI [−0.07, 0.40], *p* = 0.157).

In Model 1, White woman protagonist predicted appropriateness of emotion intensity (*b* = 0.48, 95% CI [0.09, 0.88], *p* = 0.016). In Model 2, the White woman protagonist x news engagement interaction predicted appropriateness of emotion intensity (*b* = 0.41, 95% CI [0.12, 0.70], *p* = 0.006). The effect of White woman protagonist was significant at high news engagement, *b* = 0.99, 95% CI [0.44, 1.54], *p* = 0.001, but was nonsignificant at low news engagement, *b* = 0.02, 95% CI [−0.53, 0.57], *p* = 0.946.

In Model 1, White woman protagonist predicted dispositional emotionality (*b* = −0.23, 95% CI [−0.44, −0.01], *p* = 0.041). In Model 2, the White woman protagonist x news engagement interaction predicted dispositional emotionality (*b* = −0.21, 95% CI [−0.37, −0.06], *p* = 0.008). The effect of White woman protagonist was significant at high news engagement, *b* = −0.53, 95% CI [−0.83, −0.23], *p* = 0.001, but was nonsignificant at low news engagement, *b* = 0.05, 95% CI [−0.25, 0.35], *p* = 0.741.

In Model 1, White woman protagonist did not predict competence (*b* = 0.15, 95% CI [−0.11, 0.41], *p* = 0.256). In Model 2, the White woman protagonist x news engagement interaction did not predict competence (*b* = −0.04, 95% CI [−0.24, 0.15], *p* = 0.658).

In Model 1, White woman protagonist predicted conferred status (*b* = 0.40, 95% CI [0.12, 0.68], *p* = 0.005). In Model 2, the White woman protagonist x news engagement interaction did not predict conferred status (*b* = 0.07, 95% CI [−0.14, 0.27], *p* = 0.534).

In Model 1, White woman protagonist did not predict conferred salary (*b* = 3058.86, 95% CI [−338.27, 6455.99], *p* = 0.077). In Model 2, the White woman protagonist x news engagement interaction did not predict conferred salary (*b* = 1650.73, 95% CI [−898.61, 4200.07], *p* = 0.203).

### Discussion

Supporting alternative predictions, Study 1b replicated Study 1a: White women were evaluated as more appropriate in emotion type and emotion intensity, lower in dispositional emotionality, and were conferred higher status than White men, when portrayed as angry at work. As in Study 1a, competence and conferred salary did not differ based on protagonist. Thus, we did not conceptually replicate outcomes from Brescoll and Uhlmann (2008) but instead found either the opposite pattern or no effect. Because competence was unaffected by experimental conditions across Studies 1a and 1b, we decided to omit this measure in Study 2. Conferred salary was similarly unaffected by experimental conditions across Studies 1a and 1b but was retained given the somewhat surprising findings of no differences between White women and White men in light of the gender pay gap.

News engagement affected the evaluations of appropriateness of emotional intensity and dispositional emotionality, but not appropriateness of emotion type and conferred status. News engagement moderated some of the outcomes and did so when controlling for participant’s political ideology and tendency toward socially desirable responding. News engagement, however, did not moderate some of the protagonist effects. Perhaps, more so than news engagement, the pattern of White women being evaluated more favorably than White men when angry at work was driven by a shift in beliefs that may be reflective of the great deal of attention on gender discrimination and harassment in the workplace in current news media. That is, beyond engaging with and being exposed to news on this topic, perhaps this information resulted in people endorsing the idea that women experience gender bias more so than men do at work. In Study 2, we thus explore beliefs about gendered opportunities at work as a moderator instead of news engagement *per se*.

Only for appropriateness of emotion type did invalidation affect evaluations. One possibility was that participants were focusing their attention on other features of the stimuli than on the invalidating comment. Because the emotion storyboard included the text of an email from the protagonist’s supervisor, the participants might have been focusing especially on the text rather than the primarily visual panels of the emotion storyboard. If so, participants could have been considering the specifics of the email message in their evaluation of the protagonist more so than the invalidator’s comment. Therefore, in Study 2, the panel with the email was excluded in the emotion storyboard and replaced with text indicating that the protagonist read an email before becoming angry. We thought that perhaps by making the specifics of the situation more ambiguous by omitting the message about reassignment, the invalidator’s comment would become more salient in participants’ evaluations of the protagonist.

## Study 2: the Role of Beliefs About Work Opportunities As Gendered

In Study 2, we examined whether, more so than news engagement, patterns of women being evaluated more favorably than White men were moderated by beliefs about women in the workplace during #MeToo. We reasoned that, beyond engaging with news about gender discrimination and harassment in the workplace, people also endorsed ideas that women experience bias and limited opportunity relative to men in the workplace. The specific construct of BWOG was created to capture beliefs about gender in the workplace during #MeToo.

Study 2 compared Black women and White men protagonists. We predicted that Black women would be evaluated more favorably than White men and that this effect would be moderated by BWOG, such that the effect would emerge only for those high in these beliefs. We also tested the possible role of the invalidator’s intersectional position by examining whether protagonists would be evaluated more favorably if invalidated by a White man than if invalidated by a White woman, Black woman, or Black man, and, if so, if this effect would be moderated by BWOG.

### Method

#### Participants

Workers from MTurk in the United States participated in the study online. The final sample had 297 people.

#### Materials

**Figure 3:**
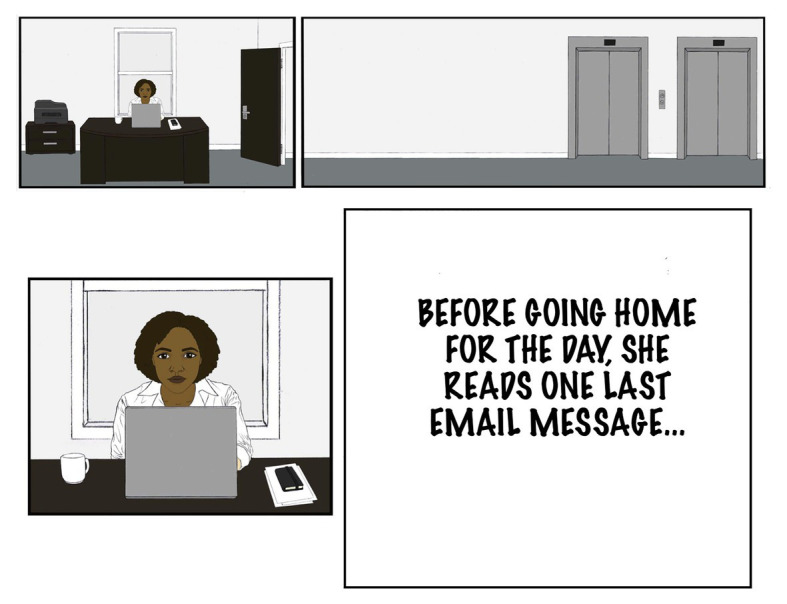
Revised panel for emotion storyboards used in Study 2.

Randomly assigned participants read one of eight emotion storyboards, identical except for intersectional position of the protagonists (Black woman or White man) and invalidators (Black woman, Black man, White woman, or White man). The emotion storyboard was the same as in Study 1b except the panel with the email text was omitted to make the situation even more ambiguous (see Figure 3).

#### Measures

Measures were identical to Studies 1a and 1b, with one addition (see Table 3 for scale reliabilities, means, and standard deviations).

*Belief in Workplace Opportunities as Gendered* (BWOG; created for this study).

BWOG was measured with a three-item scale (“Women are more likely to be passed over for assignments in the workplace than men are; Women experience more instances of bias in the workplace than men do; Men tend to get more opportunities than women do in the workplace”). Participants responded on scales ranging from 1 (Strongly disagree) to 7 (Strongly agree).

#### Procedure

The procedure was the same as in Study 1b.

### Results

For each measure, we examined the hypothesis that the effect of protagonist would be moderated by BWOG, using two regression models. Model 1 regressed measures on Black woman protagonist (dummy coded) and the contrast of being invalidated by a White man invalidator to being invalidated by others. In Model 2, we added the Black woman protagonist x BWOG interaction, as well as BWOG and the BWOG x invalidator contrast interaction to distinguish effects on measures (see Supplementary Material). For significant Black woman protagonist x BWOG interactions, we estimated simple slopes for BWOG one standard deviation above and below the mean. Simple slopes are depicted in Figure 2.

In Model 1, Black woman protagonist predicted appropriateness of emotion type (*b* = 0.34, 95% CI [0.06, 0.63], *p* = 0.019). In Model 2, the Black woman protagonist x BWOG interaction predicted appropriateness of emotion type (*b* = 0.20, 95% CI [0.01, 0.39], *p* = 0.033). The effect of Black woman protagonist was significant at high BWOG, *b* = 0.70, 95% CI [0.30, 1.10], *p* = 0.001, but was nonsignificant at low BWOG, *b* = 0.03, 95% CI [−0.36, 0.43], *p* = 0.870.

In Model 1, Black woman protagonist predicted appropriateness of emotion intensity (*b* = 0.46, 95% CI [0.12, 0.79], *p* = 0.007). In Model 2, the Black woman protagonist x BWOG interaction did not predict appropriateness of emotion intensity (*b* = 0.18, 95% CI [−0.04, 0.40], *p* = 0.112).

In Model 1, Black woman protagonist predicted dispositional emotionality (*b* = −0.37, 95% CI [−0.59, −0.15], *p* = 0.001). In Model 2, the Black woman protagonist x BWOG interaction predicted dispositional emotionality (*b* = −0.19, 95% CI [−0.33, −0.05], *p* = 0.009). The effect of Black woman protagonist was significant at high BWOG, *b* = −0.70, 95% CI [−1.01, −0.40], *p* < 0.001, but was nonsignificant at low BWOG, *b* = −0.08, 95% CI [−0.38, 0.22], *p* = 0.611.

In Model 1, Black woman protagonist predicted conferred status (*b* = 0.39, 95% CI [0.14, 0.64], *p* = 0.003). In Model 2, the Black woman protagonist x BWOG interaction predicted conferred status (*b* = 0.20, 95% CI [0.04, 0.37], *p* = 0.014). The effect of Black woman protagonist was significant at high BWOG, *b* = 0.73, 95% CI [0.38, 1.08], *p* < 0.001, but was nonsignificant at low BWOG, *b* = 0.08, 95% CI [−0.27, 0.43], *p* = 0.652.

In Model 1, Black woman protagonist did not predict conferred salary (*b* = 330.47, 95% CI [−3215.24, 3876.19], *p* = 0.855). In Model 2, the Black woman protagonist x BWOG interaction did not predict conferred salary (*b* = 1435.05, 95% CI [−868.66, 3738.76], *p* = 0.764).

### Discussion

In line with perceptions of White women in Studies 1a and 1b, Black women were evaluated as more appropriate in emotion type and intensity, lower in dispositional emotionality, and conferred more status than White men. As in Studies 1a and 1b, conferred salary did not differ based on protagonist. Thus, comparing patterns across studies, Black women and White women were evaluated similarly relative to White men.

BWOG moderated evaluations of appropriateness of emotion type, dispositional emotionality, and conferred status but not appropriateness of emotion intensity. Compared to news engagement, BWOG moderated more of the protagonist effects. We therefore examined BWOG as the moderator once more in Study 3 in our test of affirmation.

Across measures, a comment from an invalidator did not affect judgments of the protagonist, even though the invalidator may have been made more salient in this study with the omission of the email message panel. Overall, results across Studies 1a, 1b, and 2 suggest invalidation rarely affected participants’ judgments of protagonists that were angry in the workplace. Similarly, the contrast of intersectional position of the invalidator did not affect evaluations. Thus, regardless of the social group memberships of the person making the invalidating comment, invalidation did not overall negatively affect participants’ judgments of protagonists.

## Study 3: Anger Affirmation

In Study 3, we sought to replicate Study 2 findings. Additionally, we examined possible positive effects of being affirmed.

### Method

#### Participants

Workers from MTurk in the United States participated in the study online. The final sample had 296 people.

#### Materials

**Figure 4:**
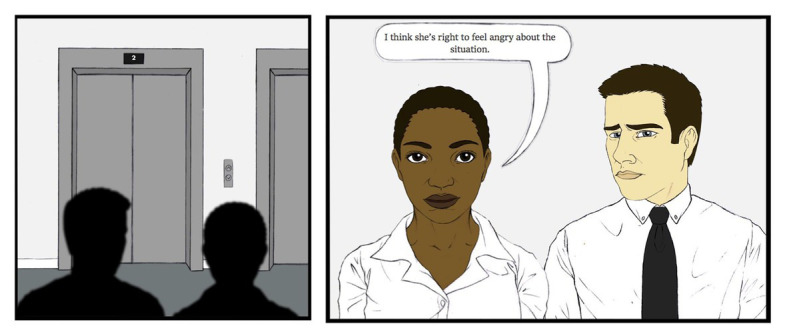
Example of last panel change for emotion storyboards used in Study 3.

Randomly assigned participants read one of six emotion storyboards, identical except for protagonists (Black woman or White man) and affirmers (Black woman, White man, or no affirmer). Emotion storyboards were identical to those from Study 2 except, for the conditions with an affirmer, the onlooker’s comment was changed from invalidation to affirmation (see Figure 4).

#### Measures

The measures were the same as in Study 2 (see Table 3).

#### Procedure

The study was hosted on MTurk and the procedure was identical to Studies 1b and 2.

### Results

For each measure, we examined the hypothesis that the effect of protagonist would be moderated by BWOG, using two regression models. For each, Model 1 regressed measures on Black woman protagonist (dummy coded) and the contrast of being affirmed to not being affirmed (coded: no affirmation = −2, affirmed by Black woman = 1, and affirmed by White man = 1). In Model 2, we added the Black woman protagonist x BWOG interaction, as well as BWOG to distinguish effects on measures (see Supplementary Material). For significant Black woman protagonist x BWOG interactions, we estimated simple slopes for BWOG one standard deviation below and above the mean. Simple slopes are depicted in Figure 2.

In Model 1, Black woman protagonist predicted appropriateness of emotion type (*b* = 0.51, 95% CI [0.25, 0.77], *p* < 0.001). The contrast of being affirmed to not being affirmed also predicted appropriateness of emotion type (*b* = 0.12, 95% CI [0.02, 0.21], *p* = 0.013). In Model 2, the Black woman protagonist x BWOG interaction predicted appropriateness of emotion type (*b* = 0.25, 95% CI [0.08, 0.43], *p* = 0.006). The effect of protagonist was significant at high BWOG, *b* = 0.79, 95% CI [0.43, 1.16], *p* < 0.001, but was nonsignificant at low BWOG, *b* = 0.11, 95% CI [−0.26, 0.48], *p* = 0.575.

In Model 1, Black woman protagonist predicted appropriateness of emotion intensity (*b* = 0.83, 95% CI [0.50, 1.15], *p* < 0.001). The contrast of being affirmed to not being affirmed also predicted appropriateness of emotion intensity (*b* = 0.17, 95% CI [0.06, 0.29], *p* = 0.003). In Model 2, the Black woman protagonist x BWOG interaction predicted appropriateness of emotion intensity (*b* = 0.32, 95% CI [0.09, 0.54], *p* = 0.006). The effect of protagonist was significant at high BWOG, *b* = 1.21, 95% CI [0.74, 1.67], *p* < 0.001, but was nonsignificant at low BWOG, *b* = 0.35, 95% CI [−0.13, 0.82], *p* = 0.149.

In Model 1, Black woman protagonist predicted dispositional emotionality (*b* = −0.72, 95% CI [−0.94, −0.51], *p* < 0.001). In Model 2, the Black woman protagonist x BWOG interaction predicted dispositional emotionality (*b* = −0.16, 95% CI [−0.31, −0.01], *p* = 0.040). The effect of protagonist was significant at high BWOG, *b* = −0.91, 95% CI [−1.22, −0.61], *p* < 0.001, and was significant at low BWOG, *b* = −0.47, 95% CI [−0.78, −0.16], *p* = 0.003.

In Model 1, Black woman protagonist predicted conferred status (*b* = 0.77, 95% CI [0.52, 1.01], *p* < 0.001). The contrast of being affirmed to not being affirmed also predicted conferred status (*b* = 0.10, 95% CI [0.01, 0.19], *p* = 0.023). In Model 2, the Black woman protagonist x BWOG interaction predicted conferred status (*b* = 0.28, 95% CI [0.10, 0.45], *p* = 0.002). The effect of protagonist was significant at high BWOG, *b* = 1.09, 95% CI [0.74, 1.45], *p* < 0.001, but was nonsignificant at low BWOG, *b* = 0.33, 95% CI [−0.03, 0.69], *p* = 0.071.

In Model 1, Black woman protagonist predicted conferred salary (*b* = 5189.61, 95% CI [1753.39, 8625.83], *p* = 0.003). In Model 2, the Black woman protagonist x BWOG interaction did not predict conferred salary (*b* = 1640.72, 95% CI [−749.97, 4031.41], *p* = 0.178).

### Discussion

Replicating Study 1a, 1b, and 2 patterns, Black women were evaluated as more appropriate in emotion type and emotion intensity, lower in dispositional emotionality, and conferred more status than White men. Unlike Studies 1a, 1b, and 2, in Study 3, Black women were also conferred higher salaries than White men.

BWOG affected evaluations of appropriateness of emotion type and intensity, dispositional emotionality, and conferred status but not salary conferral. Patterns replicated those in Study 2, with the additional moderating effect of appropriateness of emotional intensity in Study 3.

Being affirmed favorably affected evaluations of appropriateness of emotion type and emotion intensity and conferred status. Unlike invalidation in Studies 1a, 1b, and 2, affirmation positively affected participants’ evaluations of protagonists on a number of measures. The differential effect of the affirming comment compared to the invalidating comment on protagonist evaluations suggests that the nature of a comment may determine its influence on evaluations.

## General Discussion

Our results did not support the well-established expectation that representations of women’s anger would be evaluated unfavorably relative to White men’s (Lewis, 2000; Brescoll and Uhlmann, 2008; Gibson et al., 2009; Ragins and Winkel, 2011). Rather, we found that Black women and White women were judged as more appropriate and thus as more entitled to anger than White men when perceivers had strong beliefs that workplace opportunities are gendered or were high in news engagement during a time of widespread discussion of #MeToo. Our findings suggest that participants high in one or both of the measures of BWOG or news engagement may have been influenced by contemporaneous cultural events, that is, moving them to consider gender in their evaluations. Specifically, they evaluated characters within the sociocultural context that, in this case, is concerned with equity and vulnerability in the workplace. In the present studies, evaluations of anger appropriateness may suggest that participants who evaluated Black women or White women thought their protagonists were more entitled to anger than those who rated White men did due to their evaluating of anger while acknowledging the inequitable gendered reality women at work face during #MeToo.

One possibility is that participants in our studies viewed Black women’s and White women’s anger as responses to discrimination in the workplace. Because #MeToo was accessible at the time of data collection and participants did not know why the protagonist’s project was reassigned in the study manipulations, participants may have assumed the reassignment for the women protagonists happened due to discrimination. Additionally, the Project Manager was identified by a man’s name in Studies 1a and 1b and was most likely assumed to be a man even when unmarked in Studies 2 and 3 due to the higher prevalence of men than women in workplace leadership roles. Thus, participants could have interpreted the Black woman and White woman protagonist’s anger as an interpersonal response toward their supervisor or interpreted them as intergroup anger responses stemming from a gender-relevant act of discrimination (Smith and Mackie, 2015).

### Effects of Sociohistorical Context

Past studies on women’s workplace anger are not necessarily flawed; rather, we suggest that the historical context and social norms at the time of past data collection differed from those since #MeToo. Our findings add to those that have identified effects of contemporaneous cultural events on findings presumed established before (e.g., Plant et al., 2009).

Participants relatively low in BWOG or news engagement did not evaluate White men more favorably than Black or White women. This pattern may suggest that attitudes across beliefs and behavior shifted, to an extent, in the current moment in time. In line with this proposition, social group prejudices toward groups targeted by the Trump campaign in 2016 (e.g., immigrants and disabled people) were endorsed as more acceptable after Trump’s election than before by Trump and Clinton supporters alike (Crandall et al., 2018).

A follow-up attempt to replicate the present studies’ findings at a later time point in which gender attitudes may societally shift backward could directly test whether our findings reflect temporary or lasting change. Indeed, both women’s movement activity and conservative public policy shifts that simultaneously occurred during this period would predict an attitude change toward more liberal attitudes about gender (Kellstedt et al., 2010; Banaszak and Ondercin, 2016). Studies examining gender, race, and anger evaluations in the workplace, over time, are needed to determine the momentary or permanent nature of such effects. For instance, it is possible that results indicate a lasting change in the perceptions of women’s workplace anger if discrimination against women at work has become moralized (e.g., Rozin, 1999) through its widespread exposure by the #MeToo movement.

### Invalidation and Affirmation

Results also revealed that being invalidated had little effect on evaluations of the protagonist. Being affirmed, however, positively affected evaluations of anger as appropriate and the status conferred to those experiencing anger. One possibility is that affirmation may be more unusual and therefore more salient to perceivers. Perhaps invalidation is more common because people think of workplace anger as problematic (e.g., Callister et al., 2017) and thus attempt to socially regulate it by commenting on others’ displays of anger. To encourage affiliation-focused behavior, people might also be unlikely to spontaneously affirm others’ workplace anger as appropriate. If anger invalidation happens more frequently, it may be disregarded relative to affirmation due to the potential novelty of anger affirmation. Future studies should examine the range of behaviors that constitute emotion invalidation and affirmation, such as nonverbal displays or subtle comments, and how different forms could affect perceivers’ judgments.

Another possibility is that participants viewed invalidation or affirmation as confirming their assumptions that Black women and White women protagonists’ anger was a response to an experience of discrimination in the workplace from their supervisor. That is, participants may have discounted invalidation and considered affirmation in their evaluations, because it aligned with their own evaluations of protagonists’ anger during #MeToo. Affirmation in this context may have been effective because perceivers viewed the affirmation of a woman’s workplace anger as a nonaggressive confrontation of sexism (Becker and Barreto, 2014). In addition, our participants may have discounted invalidation and accentuated affirmation, because they were somewhat liberal [across Studies 1b, 2, and 3: political ideology between 3 and 4 on a scale of (1) Very Liberal to (7) Very Conservative] and relatively engaged with news [between 4 and 5 on a scale of (1) Strongly Disagree to (7) Strongly Agree regarding engagement]. Future studies could investigate if findings differ when conducted with a sample primarily or entirely disengaged with the news or conservative, given some evidence of partisan reactions to the #MeToo movement (Bucknell’s Public Policy Institute, 2018).

Additionally, the specific intersectional position of who invalidated did not affect evaluations of the protagonist. Future studies should investigate the role of relative status of the invalidator or affirmer, if direct invalidation or affirmation differs from perceiving the invalidation and affirmation of others and the downstream consequences of invalidation and affirmation in the workplace (e.g., the practice of emotion affirming at work could contribute to a more positive organizational climate).

### Limitations and Future Directions

One limitation is that we measured news engagement and beliefs about workplace opportunities as gendered in separate studies, which prevented us from examining the relationship of the constructs to one another. Therefore, we cannot conclude from our findings that the moderated effects of beliefs about opportunities as gendered necessarily indicate direct effects of the #MeToo movement. Future studies could examine if news engagement can predict beliefs about workplace opportunities as gendered by measuring these constructs in a single study and by measuring them longitudinally. Additionally, we created our beliefs about workplace opportunities as gendered measure to assess beliefs during #MeToo that people may have endorsed about gendered workplace dynamics, irrespective of their other beliefs and their own social group memberships. However, our measure may be related to validated sexism measures, such as Neosexism (Tougas et al., 1995), or be affected by participant social group memberships such as gender. Future studies should examine the relationship between these beliefs, news engagement, validated individual difference measures, and participant social group memberships.

Another limitation of the set of studies is that all relied on a design that employed the emotion storyboard. Future studies could attempt to replicate the current findings using other methods, such as video or vignette, although we know of no empirically or theoretically based reason that other equally engaging methods should yield different results. Additionally, during a time period in which gender issues were particularly accessible in popular discourse, it is possible that participants assumed the studies were about gender stereotyping and their responses were affected by experimenter demand effects. An examination of experimenter demand effects, however, found that revealing experimenter intent did not result in demand effects, even when financial incentives were offered (Mummolo and Peterson, 2019).

In our studies, we specifically examined the #MeToo movement, anger, and Black women, White women, and White men. Future directions could examine if evaluations of Black women’s anger and Black men’s anger have been similarly affected, relative to White men’s, by the #BlackLivesMatter movement. Perhaps especially so recently, during the widespread protests of Summer 2020. Additionally, future studies could test the longevity of our effect and examine if findings are specific to women’s workplace anger. For instance, pride, another gender-relevant emotion that women are expected not to express (e.g., Brosi et al., 2016) could show a similar pattern to our anger effects. Alternatively, a future study could reveal there is something unique about evaluations of women’s anger when anger is perceived as a response to workplace discrimination. Further, to our knowledge, much of the research on gender and anger does not distinguish between emotion expression and experience. Future research could parse out differences in gender and emotion research between evaluations of people described as expressing anger (e.g., information about a person’s facial expression or clenched fists) and people described as experiencing anger (e.g., “angry”).

### Implications

The most pressing question for further research is how to anticipate and track major social events that have an impact on loosely related or even seemingly unrelated phenomena we wish to study. As important, researchers must be able to determine when effects of cultural events are temporary and when a larger social change is occurring. The COVID-19 pandemic has had massive effects on how we experience and perform tasks of everyday life. The fallout of this pandemic for beliefs about and evaluations of others may change received wisdom regarding other previously established findings in ways that we have not anticipated. The challenge for researchers is to think through whether, when, and how, those effects may be made visible. Our hope for psychology is that we can be more attuned to the relation between subtle and massive shifts in everyday life and the specific questions we wish to study scientifically.

Psychologists may misinterpret unexpected findings as flukes, overlooking effects of cultural events. Often, psychologists operate with an implicit assumption that phenomena do not vary across time and context (Magnusson and Marecek, 2017). In the case of direct replication attempts, the perceived sensitivity of findings to context affects their replicability (Van Bavel et al., 2016). Our findings suggest that, outside of direct replication attempts, psychologists need to consider effects of contemporaneous cultural events and to reexamine seemingly established findings during various historical moments.

## Conclusion

Favorable evaluations of Black and White women’s workplace anger relative to White men’s, from those especially high in news engagement and BWOG, likely do not indicate that gender equality has been achieved or that such attitudes will remain stable. Rather, findings may suggest a boundary condition of stereotypes about women’s anger and point to a moment in time when large numbers of people were thinking about and discussing gender inequality in the workplace. Findings suggest urgency for psychology to consider contemporaneous cultural events in the study of stereotypes and in the discipline more broadly.

## Data Availability Statement

The raw data supporting the conclusions of this article will be made available by the authors, without undue reservation.

## Ethics Statement

The studies involving human participants were reviewed and approved by the Institutional Review Board at The Pennsylvania State University’s Office of Research Protections. Written informed consent for participation was not required for this study in accordance with the national legislation and the institutional requirements.

## Author Contributions

KM-H developed the study concept, performed testing, data collection, and data analysis, and drafted the manuscript. KM-H and SS designed the studies and interpreted the results. SS provided critical revisions. All authors contributed to the article and approved the submitted version.

### Conflict of Interest

The authors declare that the research was conducted in the absence of any commercial or financial relationships that could be construed as a potential conflict of interest.
