# Supplementary material for: Favorable Evaluations of Black and White Women’s Workplace Anger During the Era of #MeToo
Source: Front Psychol. 2021 Feb 25;12:594260. doi: 10.3389/fpsyg.2021.594260 (PMC7947812; doi:10.3389/fpsyg.2021.594260)
Supplement: Supplementary file 1 [file Table_1.docx]

Supplementary Material

# Sample Size Determinations

A priori power analyses conducted in G*Power (Faul et al., 2007) determined sample sizes to detect small to moderate effect sizes (analyses set for *d* = 0.42) with power of 0.8 for each study. Analyses revealed 222 people needed for Study 1a, 222 people needed for Study 1b, 252 people needed for Study 2, and 222 people needed for Study 3.

# Materials

Protagonists’ intersectional positions differed by gender and race. Other social group memberships were held constant that could plausibly affect evaluations, such as ability status, occupational status, socioeconomic status, and age (i.e., able-bodied, business workplace, middle-class, young). Additional social group memberships remained unmarked, such as sexual orientation, and thus participants likely assumed characters were dominant group members (e.g., heterosexual).

Gendered facial features that have been found to be confounded with emotion expression judgments (e.g., Adams, Hess, & Kleck, 2015) were held constant across protagonists, invalidators, and affirmers. To create the protagonists’ emotional expression in the final panel of the emotion storyboard, the graphic artist modeled the expression on facial expression emotion sets commonly used in psychology studies (NimStim, Tottenham et al., 2009; Pictures of Facial Affect, Friesen & Ekman, 1976). The particular expression was designed to be a high intensity anger expression (Horstmann, Lipp, & Becker, 2012).

# Materials Pilot Results

After completing a separate study, participants were asked to provide two open-ended responses to identify the gender and race of one of the characters used in the present studies. The characters used in the present studies were all identified by participants with over 80% consensus. For participants who saw: the Black woman protagonist, (*N* = 45) 100% classified her as woman/female, 100% described her race as Black/African-American; the White woman protagonist, (*N* = 49) 100% classified her as woman/female, 98% described her race as White/Caucasian/European; the White man protagonist, (*N* = 49) 100% classified him as man/male, 98% described his race as White/Caucasian/European; the Black man invalidator/affirmer, (*N* = 51) 96% classified him as man/male, 98% described his race as Black/African-American.

In a subsequent pilot study, participants identified one of the remaining invalidators/affirmers. For the invalidator/affirmer identifications, participants asked about: the White man invalidator/affirmer, (*N* = 22) 100% classified him as man/male, 86% described his race as White/Caucasian; the Black woman invalidator/affirmer, (*N* = 22) 100% classified her as woman/female, 100% described her race as Black/African-American; the White woman invalidator/affirmer, (*N* = 19) 100% classified her as woman/female, 100% described her race as White/Caucasian/European.

These same participants also rated the emotion of one of the protagonists, as pictured in the final panel without the accompanying panel text and depicted expressing anger outside of the larger emotion storyboard. Piloting was conducted to ensure facial expressions of emotion were rated as more than moderately angry (higher than 4 on a scale of Not angry at all (1) to Very angry (7). This rating metric was chosen to reflect the likelihood of perceptual overlap among facial expressions when presented in a decontextualized format (e.g., Aviezer et al., 2008). Participants rated the protagonists on a number of emotions, each on scales from Not at all (1) to Very (7). Ratings for the Black woman protagonist (*N* = 20) were the following: angry *M* = 6.00, *SD* = 1.26; disgusted *M* = 5.15, *SD* = 1.42; surprised *M* = 2.80, *SD* = 1.61; sad *M* = 2.75, *SD* = 1.65; afraid *M* = 2.60, *SD* = 1.67; happy *M* = 1.55, *SD* = 1.23. Ratings for the White woman protagonist (*N* = 19) were the following: angry *M* = 6.79, *SD* = 0.54; disgusted *M* = 5.11, *SD* = 2.03; surprised *M* = 2.53, *SD* = 1.68; afraid *M* = 2.26, *SD* = 1.59; sad *M* = 1.89, *SD* = 1.29; happy *M* = 1.05, *SD* = 0.23. Ratings for the White man protagonist (*N* = 19) were the following: angry *M* = 6.58, *SD* = 0.84; disgusted *M* = 5.42, *SD* = 1.84; surprised *M* = 3.00, *SD* = 2.08; sad *M* = 2.32, *SD* = 1.64; afraid *M* = 1.74, *SD* = 1.15; happy *M* =. 1.11, *SD* = 0.32.

# Items for Study 1a Measures

### Appropriateness of emotion type (emotion type present subscale of the Perceived Emotional Appropriateness Scale; Warner & Shields, 2009)

1. The emotions displayed by the main character were wrong. (reverse-coded, R)

2. I would not have shown the types of emotions that the main character displayed. (R)

3. The main character's emotions were exactly the kinds that were called for.

4. I think the types of emotions that the main character felt were normal.

### Appropriateness of emotion intensity (emotional intensity subscale of the Perceived Emotional Appropriateness Scale; Warner & Shields, 2009)

1. The main character was too emotional. (R)

2. Most people would not have been so emotional at certain points as the main character was. (R)

3. I think that the main character had too much emotion for clear thinking. (R)

4. The emotions shown by the main character were too extreme. (R)

5. I think the main character was emotionally out of control. (R)

### Dispositional emotionality (adapted from Brescoll & Uhlmann, 2008 and McCormick-Huhn, Zawadzki, & Shields, in prep.)

1. In general, how emotional is the main character?

2. In general, how defensive is the main character?

3. In general, how likely is the main character to overreact?

4. The main character became angry because of his or her personality.

5. The main character became angry because he or she is an angry person.

6. The main character became angry because of the situation with his or her boss. (R)

### Authenticity (adapted from Zawadzki, Warner, & Shields, 2013)

1. How authentic was the main character's emotion?

2. How genuine was the main character's emotion?

3. How much did the main character experience deep feelings?

4. How fake was the main character's emotion? (R)

### Competence (adapted from Brescoll & Uhlmann, 2008)

1. How competent is the main character?

2. How skilled is the main character?

### Conferred status (adapted from Tiedens, 2001)

1. How much status does the main character deserve?

2. How much power does the main character deserve?

3. How much independence does the main character deserve?

4. How likely would you be to hire the main character?

### Conferred salary (adapted from Brescoll & Uhlmann, 2008; open-ended)

In the text box below please type the yearly salary amount you would pay the main character.

# Study 1a Results

Authenticity did not differ by protagonist (*p* = .068; White woman protagonist *M=* 5.55, *SD* = 0.86; White man protagonist *M* = 5.32, *SD* = 0.99), invalidator (*p* = .239) or the interaction of protagonist and invalidator (*p* = .729).

# Study 1b Measures

### Appropriateness of emotion type (same items as Study 1a)

### Appropriateness of emotion intensity (same items as Study 1a)

### Dispositional emotionality (same items as Study 1a)

### Competence (same items as Study 1a)

### Conferred status (same items as Study 1a)

### Conferred salary (same item as Study 1a)

### News Engagement Scale (moderator; created for this study)

1. To what extent are you familiar with the #MeToo movement?

2. How frequently do you read news articles?

3. How often have you come across news articles about gender discrimination in the workplace?

### Marlowe–Crowne Social Desirability Scale 13-Item Short Form (Reynolds, 1982; participants responded by selecting if the described behavior was “true” or “false” of themselves)

1. It is sometimes hard for me to go on with my work if I am not encouraged. (R)

2. I sometimes feel resentful when I don't get my own way. (R)

3. On a few occasions, I have given up doing something because I thought too little of my ability. (R)

4. There have been times when I felt like rebelling against people in authority even though I knew they were right. (R)

5. No matter who I’m talking to, I’m always a good listener.

6. There have been occasions when I took advantage of someone. (R)

7. I’m always willing to admit it when I make a mistake.

8. I sometimes try to get even, rather than forgive and forget. (R)

9. I am always courteous, even to people who are disagreeable.

10. I have never been irked when people expressed ideas very different from my own.

11. There have been times when I was quite jealous of the good fortune of others. (R)

12. I am sometimes irritated by people who ask favors of me. (R)

13. I have never deliberately said something that hurt someone’s feelings.

# Study 2 Measures

### Appropriateness of emotion type (same items as Study 1a)

### Appropriateness of emotion intensity (same items as Study 1a)

### Dispositional emotionality (same items as Study 1a)

### Conferred status (same items as Study 1a)

### Conferred salary (same item as Study 1a)

### Belief in Workplace Opportunities as Gendered (moderator; created for this study)

1. Men tend to get more opportunities than women do in the workplace.

2. Women experience more instances of bias in the workplace than men do.

3. Women are more likely to be passed over for assignments in the workplace than men are

# Study 3 Measures

### Appropriateness of emotion type (same items as Study 1a)

### Appropriateness of emotion intensity (same items as Study 1a)

### Dispositional emotionality (same items as Study 1a)

### Conferred status (same items as Study 1a)

### Conferred salary (same item as Study 1a)

### Belief in Workplace Opportunities as Gendered (same items as Study 2)

# Salary Exclusions

For conferred salary, outliers as determined by boxplots, and people who did not provide amounts, were excluded from analysis for each study: 12 from Study 1a, four from Study 1b, five from Study 2, and nine from Study 3.

# Study 1b Predictor Coding and Results

Model 1: White woman protagonist (dummy coded: 0 = White man, 1 = White woman), contrast of being invalidated to not being invalidated (coded: no invalidation = -2, invalidated by White woman = 1, invalidated by White man =1), contrast of being invalidated by a White man invalidator to being invalidated by a White woman (coded: no invalidation = 0, invalidated by White woman = 1, invalidated by White man = -1), interaction of invalidator and protagonist, and demographic variables of political ideology (mean-centered) and tendency toward socially desirable responding (mean-centered). Model 2 added: White woman protagonist x news engagement interaction and news engagement (news engagement variable mean-centered).

Participants’ news engagement was correlated with their political ideology (*r* = -0.30, *p* < .001), such that higher liberalism was moderately associated with higher news engagement. Participants’ news engagement was not correlated with their tendency toward socially desirable responding (*r* = -0.03, *p* = .606).

## Supplementary Table for Study 1b

|  | | | | | | | | | | | | | |  |
| --- | --- | --- | --- | --- | --- | --- | --- | --- | --- | --- | --- | --- | --- | --- |
| **Supplementary Table 1.** Study 1b unstandardized regression coefficients of predictors, split by measure. | | | | | | | | | | | | | |  |
|  |  | Model 1 | | | | | |  | Model 2 | | | | | |
|  | Measure | Appro.Type | Appro. Inten | Dispo. E. | Competence | Status | Salary |  | Appro.Type | Appro. Inten | Dispo. E. | Competence | Status | Salary |
| Predictor |  |  |  |  |  |  |  |  |  |  |  |  |  |  |
| WW P | | 0.46* | 0.48* | -0.23* | 0.15 | 0.40* | 3058.86 |  | 0.45* | 0.48* | -0.23* | 0.18 | 0.41* | 2983.49 |
| No invalidator | | -0.12* | -0.06 | 0.01 | -0.06 | -0.02 | 651.51 |  | -0.12* | -0.07 | 0.03 | -0.06 | -0.02 | 637.98 |
| WM to WW invalidator | | -0.12 | -0.20 | 0.11 | -0.03 | -0.01 | 1854.66 |  | -0.12 | -0.22 | 0.13 | -0.01 | -0.004 | 1900.72 |
| WW P x WM to WW invalidator | | 0.15 | -0.02 | 0.03 | 0.24 | -0.05 | -816.89 |  | 0.13 | -0.04 | 0.03 | 0.23 | -0.05 | -1298.07 |
| News engagement | | -- | -- | -- | -- | -- | -- |  | -0.03 | -0.23* | 0.17* | 0.08 | -0.03 | 551.49 |
| WW P x News engagement | | -- | -- | -- | -- | -- | -- |  | 0.17 | 0.41* | -0.21* | -0.04 | 0.07 | 1650.73 |
| Political ideology | | -- | -- | -- | -- | -- | -- |  | 0.07 | 0.03 | 0.03 | 0.06 | 0.01 | 242.42 |
| Social desirability | | -- | -- | -- | -- | -- | -- |  | -0.52 | -0.31 | -0.14 | 0.51 | 0.32 | 1945.28 |
|  |  |  |  |  |  |  |  |  |  |  |  |  |  |  |
| Constant |  | 4.60** | 3.93** | 4.26** | 4.52** | 3.82** | 43576.23** |  | 4.60** | 3.91** | 4.27** | 4.51** | 3.81** | 43540.75** |
|  |  |  |  |  |  |  |  |  |  |  |  |  |  |  |
| *F*(degrees of  freedom) | | 3.69  (4, 263)* | 2.38  (4, 263) | 1.92  (4, 262) | 1.54  (4, 263) | 2.1  (4, 263) | 1.47  (4, 259) |  | 2.53  (8, 259)* | 2.21  (8, 259)* | 2.33  (8, 258)* | 1.62  (8, 259) | 1.27  (8, 259) | 1.6  (8, 255) |
| *R^2^* |  | 0.053 | 0.035 | 0.028 | 0.023 | 0.031 | 0.022 |  | 0.072 | 0.064 | 0.067 | 0.048 | 0.038 | 0.048 |

Note: * *p* < .05; ** *p* < .001. WW P indicates White woman protagonist, No invalidator indicates contrast of being invalidated to not, WM to WW invalidator indicates contrast of White man invalidator to White woman invalidator, Appro. Type indicates appropriateness of emotion type, Appro. Inten. indicates appropriateness of emotion intensity, and Dispo. E. indicates dispositional emotionality.

# Study 2 predictor Coding and Results

Model 1: Black woman protagonist (dummy coded: 0 = White man, 1= Black woman) and contrast of being invalidated by a White man invalidator to being invalidated by others (dummy coded: 0 = White woman, 0 = Black woman, 0 = Black man, 1= White man). Model 2 added: Black woman protagonist x BWOG interaction, BWOG, and BWOG x invalidator contrast interaction (BWOG variable centered).

## Supplementary Table for Study 2

| **Supplementary Table 2.** Study 2 unstandardized regression coefficients of predictors, split by measure. | | | | | | | | | | | |  |
| --- | --- | --- | --- | --- | --- | --- | --- | --- | --- | --- | --- | --- |
|  |  | Model 1 | | | | |  | Model 2 | | | | |
|  | Measure | Appro.Type | Appro. Inten | Dispo. E. | Status | Salary |  | Appro.Type | Appro. Inten | Dispo. E. | Status | Salary |
| Predictor |  |  |  |  |  |  |  |  |  |  |  |  |
| BW P | | 0.34* | 0.46* | -0.37* | 0.39* | 330.47 |  | 0.34* | 0.45* | -0.37* | 0.39* | 407.36 |
| WM invalidator | | 0.19 | 0.28 | -0.21 | 0.16 | -2527.1 |  | 0.13 | 0.24 | -0.17 | 0.11 | -2965.85 |
| BWOG | | -- | -- | -- | -- | -- |  | -0.02 | -0.09 | 0.12* | -0.04 | 265.64 |
| BW P x BWOG | | -- | -- | -- | -- | -- |  | 0.20* | 0.18 | -0.19* | 0.20* | 1435.05 |
| BWOG x WM invalidator | | -- | -- | -- | -- | -- |  | 0.11 | 0.11 | -0.06 | 0.01 | 395.84 |
|  |  |  |  |  |  |  |  |  |  |  |  |  |
| Constant |  | 4.73** | 4.21** | 4.23** | 4.00** |  |  | 4.75** | 4.22** | 4.22** | 4.01** | 46860.94** |
|  |  |  |  |  |  |  |  |  |  |  |  |  |
| *F*(degrees of freedom) | | 3.76  (2, 294)* | 5.09  (2, 294)* | 7.62  (2, 294)* | 5.62  (2, 294)* | 0.74  (2, 289) |  | 3.81  (5, 291)* | 2.84  (5, 291)* | 4.68  (5, 291)** | 4.07  (5, 291)* | 1.31  (5, 286) |
| *R^2^* |  | 0.025 | 0.033 | 0.049 | 0.037 | 0.005 |  | 0.061 | 0.046 | 0.074 | 0.065 | 0.022 |

Note: * *p* < .05; ** *p* < .001. BW P indicates Black woman protagonist, WM invalidator indicates contrast of White man invalidator to all other invalidators, BWOG indicates beliefs about workplace opportunities as gendered, Appro. Type indicates appropriateness of emotion type, Appro. Inten. indicates appropriateness of emotion intensity, and Dispo. E. indicates dispositional emotionality.

# Study 3 predictor Coding and Results

Model 1: Black woman protagonist (dummy coded: 0 = White man, 1= Black woman) and contrast of being affirmed to not being affirmed (coded: no affirmation = -2, affirmed by Black woman = 1, affirmed by White man = 1). Model 2 added: Black woman protagonist x BWOG interaction and BWOG (BWOG variable centered).

## Supplementary Table for Study 3

|  | | | | | | | | | | | |  |
| --- | --- | --- | --- | --- | --- | --- | --- | --- | --- | --- | --- | --- |
| **Supplementary Table 3.** Study 3 unstandardized regression coefficients of predictors, split by measure. | | | | | | | | | | | |  |
|  |  | Model 1 | | | | |  | Model 2 | | | | |
|  | Measure | Appro.Type | Appro. Inten | Dispo. E. | Status | Salary |  | Appro.Type | Appro. Inten | Dispo. E. | Status | Salary |
| Predictor |  |  |  |  |  |  |  |  |  |  |  |  |
| BW P | | 0.51** | 0.83** | -0.72** | 0.77** | 5189.61* |  | 0.46** | 0.80** | -0.69** | 0.72** | 4882.06* |
| Affirmer | | 0.12* | 0.17* | -0.02 | 0.1* | 940.67 |  | 0.13* | 0.19* | -0.03 | 0.11* | 1017.4 |
| BWOG | | -- | -- | -- | -- | -- |  | -0.04 | -0.09 | 0.02 | -0.06 | -116.57 |
| BW P x BWOG | | -- | -- | -- | -- | -- |  | 0.25* | 0.32* | -0.16* | 0.28* | 1640.72 |
|  |  |  |  |  |  |  |  |  |  |  |  |  |
| Constant |  | 5.03** | 4.4** | 4.14** | 3.97** | 44763.73** |  | 5.02** | 4.38** | 4.15** | 3.95** | 44729.79** |
|  |  |  |  |  |  |  |  |  |  |  |  |  |
| *F*(degrees of freedom) | | 10.23  (2, 293)** | 16.23  (2, 293)** | 21.65  (2, 293)** | 20.07  (2, 292)** | 5.83  (2, 284)* |  | 8.11  (4, 291)** | 10.47  (4, 291)** | 12.52  (4, 291)** | 13.40  (4, 290)** | 3.450  (4, 282)* |
| *R^2^* |  | 0.065 | 0.100 | 0.129 | 0.121 | 0.037 |  | 0.100 | 0.126 | 0.147 | 0.156 | 0.047 |

Note: * *p* < .05; ** *p* < .001. BW P indicates Black woman protagonist, affirmer indicates contrast of being affirmed to not, BWOG indicates beliefs about workplace opportunities as gendered, Appro. Type indicates appropriateness of emotion type, Appro. Inten. indicates appropriateness of emotion intensity, and Dispo. E. indicates dispositional emotionality.

# References

Adams, R. B., Hess, U., & Kleck, R. E. (2015). The intersection of gender-related facial appearance and facial displays of emotion. *Emotion Review, 7*, 5-13. doi: 10.1177/1754073914544407

Aviezer, H., Hassin, R. R., Ryan, J., Grady, C., Susskind, J., Anderson, A., ... & Bentin, S. (2008). Angry, disgusted, or afraid? Studies on the malleability of emotion perception. *Psychological Science*, *19*, 724-732.

Faul, F., Erdfelder, E., Lang, G., Buchner A. (2007). G* Power 3: A flexible statistical power analysis program for the social, behavioral, and biomedical sciences. *Behavioral Research Methods.* *39*, 175-91.

Horstmann, G., Lipp, O. V., & Becker, S. I. (2012). Of toothy grins and angry snarls—Open mouth displays contribute to efficiency gains in search for emotional faces. *Journal of Vision*, *12*, 1-15. doi:10.1167/12.5.7

Tottenham, N., Tanaka, J. W., Leon, A. C., McCarry, T., Nurse, M., Hare, T. A., ... & Nelson, C. (2009). The NimStim set of facial expressions: judgments from untrained research participants. *Psychiatry Research*, *168*, 242-249. doi:10.1016/j.psychres.2008.05.006
